# Preparation and Epitope Identification of Monoclonal Antibodies Against African Swine Fever Virus pE120R

**DOI:** 10.3390/vetsci13040358

**Published:** 2026-04-07

**Authors:** Juan Li, Miaomiao Ye, Peng Gao, Yajin Qu, Quanlin Li, Qiongqiong Zhou, Yongning Zhang, Lei Zhou, Xinna Ge, Xin Guo, Jun Han, Hanchun Yang

**Affiliations:** 1 State Key Laboratory of Veterinary Public Health Safety, College of Veterinary Medicine, China Agricultural University, Beijing 100193, China; s20233051008@cau.edu.cn (J.L.); yemiaomiao1996@163.com (M.Y.); quyajin@cau.edu.cn (Y.Q.); sweetquanlin@cau.edu.cn (Q.L.); zhouqq@cau.edu.cn (Q.Z.); zhangyongning@cau.edu.cn (Y.Z.); leosj@cau.edu.cn (L.Z.); gexn@cau.edu.cn (X.G.); guoxincau@cau.edu.cn (X.G.); hanx0158@cau.edu.cn (J.H.); yanghanchun1@cau.edu.cn (H.Y.); 2 Key Laboratory of Animal Epidemiology of Ministry of Agriculture and Rural Affairs, College of Veterinary Medicine, China Agricultural University, Beijing 100193, China

**Keywords:** African swine fever virus, capsid protein pE120R, monoclonal antibody, B-cell epitope

## Abstract

The capsid protein pE120R of African swine fever virus (ASFV) is highly immunogenic and is thought to play an important role in viral replication, yet its molecular characteristics and functions during infection remain poorly understood. Here, we generated two monoclonal antibodies (mAbs), 1C11 and 3G7, against ASFV pE120R and characterized their specificity and utility. Epitope mapping showed that 1C11 recognized the linear epitope ^109^KKHLFP^114^, whereas 3G7 recognized ^112^LFPKL^116^. These antibodies enabled analysis of pE120R expression and localization during ASFV infection, demonstrating that pE120R is expressed at a late stage and partially co-localizes with the structural protein p54 in viral factories. Together, these results provide valuable immunological tools for further investigation of pE120R in ASFV replication and pathogenesis.

## Simple Summary

As the primary threat to the global pig industry, ASFV is a giant DNA virus encoding numerous proteins that are of unknown function yet essential for viral replication and virulence. Here, we developed two monoclonal antibodies targeting the replication-essential protein pE120R and mapped its linear epitopes. Notably, these epitopes are surface-accessible and highly conserved among ASFV strains. This work offers potent tools for investigating pE120R’s role in the ASFV life cycle and contributes to a deeper understanding of viral pathogenesis.

## 1. Introduction

African swine fever (ASF) is an acute and highly contagious disease of domestic pigs and wild boars caused by African swine fever virus (ASFV). Because of its high lethality, rapid spread, and major economic consequences, ASF continues to pose a serious threat to the global swine industry [1]. First reported in Kenya in 1921, ASFV spread to Eastern Europe in 2007 [2], subsequently reaching Asia and causing major outbreaks in China in 2018 [3,4]. The emergence of recombinant genotype I/II strains has further complicated disease control [5]. Currently, the lack of safe and effective vaccines or specific antiviral treatments continues to challenge ASF control efforts [6,7].

As a nucleocytoplasmic large DNA virus with particles approximately 200 nm in diameter, ASFV has a multilayered structure consisting of an outer envelope, envelope, inner envelope, core shell and nucleoid [8,9]. Its genome spans 170–190 kb and encodes over 150 proteins, of which about 50 are incorporated into mature virions [10,11]. Both structural and nonstructural proteins are known to participate in viral replication, assembly, transport, and immune modulation [12]; however, the functions of a considerable number of ASFV proteins remain incompletely understood, and the lack of specific research tools has limited detailed investigation of their molecular characteristics and roles during infection.

pE120R, also known as p14.5, is a late-expressed structural protein encoded by the ASFV *E120R* gene and is considered to be associated with the capsid or virion [13]. Previous studies have shown that pE120R contributes to virion assembly and intracellular transport through interactions with the major capsid protein p72 and host cytoskeletal components [14,15]. In addition, pE120R undergoes N-terminal alanine acetylation during infection, a modification that may be relevant to the ASFV life cycle, given that many viruses exploit acetylation-mediated signaling to regulate viral protein function [16]. Meanwhile, pE120R has also been implicated in immune regulation. It suppresses type I interferon (IFN-β) induction by targeting IRF3 and STING phosphorylation and inhibits NF-κB nuclear translocation [17,18]. Deletion of *E120R* enhances innate immune activation and impairs viral release [19]. Collectively, these studies suggest that pE120R is important for efficient ASFV replication. However, despite these functional insights, its antigenic properties and expression characteristics during ASFV infection remain poorly understood, and specific immunological tools for its investigation are still lacking.

In this study, we generated monoclonal antibodies against pE120R using a recombinant protein-based immunization strategy. We further characterized their reactivity, defined the linear epitopes they recognize, and applied them to analyze the expression pattern and subcellular localization of pE120R during ASFV infection. These antibodies provide useful tools for investigating the biological role of pE120R in ASFV replication and pathogenesis.

## 2. Materials and Methods

### 2.1. Cells, Viruses, and Experimental Animals

The fourth-round subclonal cell line derived from the wild boar lung cell line (WSL-R4) and primary porcine alveolar macrophages (PAMs) isolated from 1-month-old specific-pathogen-free (SPF) piglets were maintained in RPMI-1640 medium supplemented with 10% fetal bovine serum (FBS; Gibco, Thermo Fisher Scientific, Waltham, MA, USA). SP2/0 myeloma cells were cultured in Dulbecco’s Modified Eagle’s Medium (DMEM; Gibco, Thermo Fisher Scientific, Waltham, MA, USA) supplemented with 20% FBS. The genotype II ASFV strain CADC_HN09 (ASFV-HN09) (GenBank accession no. MZ614662.1) was used as the model pathogen in this study. All ASFV-related procedures were performed in strict accordance with the biosafety regulations of the Biosafety Level 3 (BSL-3) laboratory at China Agricultural University. BALB/c mice were obtained from SPF (Beijing) Biotechnology Co., Ltd (Beijing, China).

### 2.2. Construction of Recombinant Plasmids Containing the ASFV E120R Gene

To facilitate antibody production and detection, as well as epitope mapping, multiple full-length and truncated E120R fragments were constructed and amplified in this study. Specific primers (Table 1) were designed based on the E120R gene sequence of ASFV CADC_HN09 (GenBank accession no. MZ614662.1), according to the strategy for generating full-length and truncated constructs, with the aid of SnapGene (version 6.0.2) for in silico primer design and evaluation. PCR amplification was performed using the genomic DNA of the ASFV HN09 strain as the template. A common PCR protocol was applied to all primer sets using KOD One PCR Master Mix (TOYOBO, Osaka, Japan). Each reaction was carried out in a total volume of 50 μL, containing 50 ng of genomic DNA, 15 pmol of each of the forward and reverse primers, 25 μL of KOD One PCR Master Mix, and sterile water to bring the final volume of 50 μL. The amplification conditions were as follows: 98 °C for 10 s, (Tm − 5) °C for 5 s, and 68 °C for 5 s/kb, for 30 cycles. The amplified products were inserted into the prokaryotic expression vector pGEX-6p-1 and the eukaryotic expression vector pEGFP-C1 via homologous recombination to generate recombinant plasmids pGEX-6p-1-E120R and pEGFP-E120Rs.

### 2.3. Expression and Purification of ASFV pE120R in Prokaryotic Cells

The pGEX-6p-1-E120R plasmid was transformed into competent *E. coli* Rosetta (DE3) cells and plated on LB agar containing ampicillin and chloramphenicol, followed by overnight incubation at 37 °C. A single colony was inoculated into LB medium containing the same antibiotics and cultured with shaking to a final volume of 200 mL at 37 °C until OD_600_ reached 0.6–0.8. Protein expression was induced by adding 0.1 mM IPTG and incubating at 16 °C for 18 h. Cells were harvested by centrifugation at 5000 rpm for 5 min, resuspended in PBS, and washed by repeated centrifugation. Cells were lysed by sonication on ice at 26% amplitude with a cycle of 5 s on and 5 s off for 30 min, and then centrifuged at 8000 rpm for 10 min to separate the supernatant and pellet. Expression was assessed by SDS-PAGE and confirmed by Western blot. GST-pE120R fusion protein was purified using glutathione–agarose resin (Cytiva, Marlborough, MA, USA) and eluted with buffer containing 50 mM Tris-HCl (pH 8.0) and 10 mM reduced glutathione. The eluted proteins were further verified by SDS-PAGE and Western blot.

### 2.4. Expression of ASFV pE120R and Its Truncated Forms in Eukaryotic Cells

WSL-R4 cells were seeded in cell culture plates or on coverslips and transfected with pcDNA3.1-E120R-myc or pEGFP-C1-E120Rs plasmids using Lipofectamine 2000 Reagent (Invitrogen, Thermo Fisher Scientific, Waltham, MA, USA) when they reached 80–90% confluence to express recombinant proteins in eukaryotic cells. At 24 h post-transfection, the cells were fixed with 4% paraformaldehyde, and protein expression was evaluated by indirect immunofluorescence assay (IFA).

### 2.5. Preparation of Monoclonal Antibodies Against ASFV pE120R

Purified GST-pE120R protein was used as an immunogen to immunize four 6–8-week-old female BALB/c mice. For the primary immunization, the antigen emulsified with Freund’s complete adjuvant was administered subcutaneously at multiple sites on the back. Booster immunizations were performed twice at 2-week intervals using the antigen emulsified with Freund’s incomplete adjuvant via intramuscular injection, with each mouse receiving 50 μg antigen per immunization. Seven days after the third immunization, blood was collected from the orbital venous plexus to obtain serum, and antibody titers were determined by IFA under both transfection and infection conditions. The mouse with the highest titer received a final intraperitoneal booster of 50 μg of GST-pE120R without adjuvant. Peritoneal macrophages from two BALB/c mice were prepared as feeder cells and plated into 96-well plates one day in advance. Three days after the final booster, splenocytes were isolated from the immunized mouse and fused with SP2/0 myeloma cells at a ratio of 5:1 using polyethylene glycol solution (PEG; average mol wt 1450; Sigma-Aldrich, St. Louis, MO, USA) at 37 °C. Fused cells were cultured in HAT selection medium. After 10–14 days, supernatants were screened by IFA for pE120R-specific antibodies. Positive hybridomas were expanded in HT medium and subcloned three times by limiting dilution to obtain stable monoclonal antibody-secreting cell lines [20].

For ascites production, each mouse was intraperitoneally injected with 0.5 mL of Freund’s incomplete adjuvant 3 days before hybridoma inoculation. Hybridoma cells in the logarithmic growth phase were collected, centrifuged at 1000 rpm for 5 min, washed twice with sterile PBS, counted, and resuspended in sterile PBS to a final density of 2 × 10^6^ cells/mL. Each mouse was then intraperitoneally injected with 0.5 mL of the cell suspension. Ascites usually developed 7–14 days later. When mice showed obvious abdominal distension, reduced activity, and ruffled fur, ascitic fluid was collected. Briefly, the mouse was restrained, the abdominal skin was stretched, and an 18 G needle was inserted approximately 1 cm into the lower left or lower right abdomen. The ascitic fluid was allowed to drain naturally into a 15 mL centrifuge tube. The collected ascites was centrifuged at 1000 rpm for 10 min at room temperature to remove cell debris and clots. The supernatant was then collected, aliquoted, and stored at −80 °C until use.

### 2.6. Indirect Immunofluorescence Assay (IFA)

WSL-R4 cells were seeded in 96-well plates and subjected to plasmid transfection or viral infection. For transfection, cells at 80–90% confluence were transfected with pcDNA3.1-E120R-myc plasmid. For infection, confluent cells were infected with ASFV at an MOI of 0.1. After 24 h, cells were fixed with cold 4% paraformaldehyde for 15 min, washed with PBS, permeabilized with 0.1% Triton X-100 for 30 min, and blocked with 1% BSA. For transfected samples, rabbit anti-Myc antibody and serially diluted mouse serum (diluted from 1:500 to 1:4000 in a twofold manner) or monoclonal antibodies were used as primary antibodies; for infected samples, rabbit anti-ASFV p54 antibody and mouse antibodies were used. After incubation and washing, fluorescent secondary antibodies were applied. Nuclei were stained with DAPI, and cells were imaged under a fluorescence microscope. A sample was considered positive when specific fluorescence signals were observed in the transfected or infected group, whereas no specific signal was detected in the negative control group. For each well, 3–5 random fields were examined, and all IFA assays were repeated in three independent experiments.

For temporal localization analysis, PAMs and WSL-R4 cells grown on coverslips were infected with ASFV (an MOI of 0.1) and fixed at 0, 6, 12, and 18 hpi. After IFA staining and mounting with antifade medium, samples were imaged using a Nikon A1 confocal microscope (Nikon Instruments Inc., Melville, NY, USA) and analyzed with NIS-Elements (version 5.21.00).

### 2.7. Western Blot

Standard SDS-PAGE gels were prepared. WSL-R4 cells were transfected with pEGFP-C1-E120R plasmid or infected with ASFV, and total proteins were extracted using NP-40 lysis buffer at 24 h post-treatment or post-infection. Samples were mixed with loading buffer, boiled at 100 °C for 5–10 min, and electrophoresed (80 V initial voltage, then increased to 120 V). Proteins were transferred to PVDF membranes using wet transfer (100 V, 120 min). Membranes were blocked with 5% skim milk for 2 h and washed with PBST. Primary antibody incubation was performed overnight at 4 °C, followed by HRP-conjugated secondary antibody incubation (ZSGB-BIO, Beijing, China). Signals were detected using ECL reagent and captured with a chemiluminescence imaging system. A signal was considered positive when a clear immunoreactive band at the expected molecular weight was detected in the transfected or infected sample but not in the corresponding negative control. Western blot analyses of both transfected and infected samples were performed in three independent experiments.

### 2.8. Isotype Identification of Monoclonal Antibodies

The isotypes of the obtained monoclonal antibodies were determined using a Mouse Monoclonal Antibody Isotyping Kit (Proteintech Group, Rosemont, IL, USA) according to the manufacturer’s instructions. Briefly, the pE120R monoclonal antibodies were added to wells pre-coated with subtype-specific anti-mouse immunoglobulin antibodies for the detection of IgG1, IgG2a, IgG2b, IgG3, IgM, and IgA. After incubation at room temperature for the recommended time, the corresponding enzyme-conjugated secondary antibody was added and incubated further. After washing, the substrate solution was added for color development. The immunoglobulin isotype of each monoclonal antibody was determined according to the colorimetric results.

## 3. Results

### 3.1. Expression and Purification of the pE120R Protein

**Figure 1:**
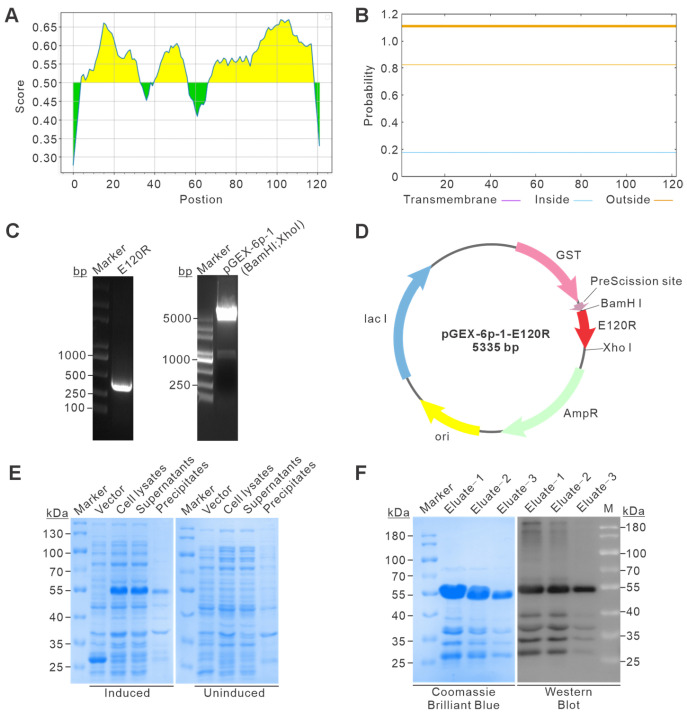
Expression and purification of the pE120R protein. (**A**) Prediction of linear B-cell epitopes in pE120R via using the Immune Epitope Database analysis tool (http://tools.immuneepitope.org/bcell/ (accessed on 15 June 2024)). Using 0.50 as the threshold, yellow regions indicate residues with scores above the threshold, suggesting a higher probability of forming β-turns and potential antigenicity, whereas green regions indicate residues with scores below the threshold. (**B**) Prediction of the transmembrane topology of pE120R using the TMHMM online tool. (**C**) Amplification of the *E120R* gene and double-enzyme digestion of the pGEX-6p-1 vector. (**D**) Schematic representation of the recombinant plasmid pGEX-6p-1-E120R. (**E**) SDS-PAGE analysis of GST-pE120R expression in *Escherichia coli*. (**F**) Purification of GST-pE120R protein. Eluted fractions were analyzed by SDS-PAGE followed by Coomassie Brilliant Blue staining (**left**) and Western blot analysis using an anti-GST antibody (**right**).

To preliminarily assess the antigenic potential of pE120R, an in silico analysis was first performed to predict potential B-cell epitopes. The prediction profile showed multiple regions with scores above the threshold, suggesting that pE120R contains several potentially immunoreactive segments and supporting its suitability as an antigen for monoclonal antibody generation (Figure 1A). These regions may represent potential surface-accessible and immunogenic motifs that contribute to antibody recognition. Transmembrane topology analysis further showed that no significant transmembrane helices were present in pE120R, suggesting that the protein is unlikely to be a membrane-spanning protein (Figure 1B). Taken together, these features are consistent with its proposed localization as a capsid-associated structural protein, and support the accessibility of its antigenic regions to antibody binding.

To obtain a recombinant antigen for antibody production, the ASFV *E120R* gene was amplified and successfully cloned into the pGEX-6p-1 vector. Agarose gel electrophoresis showed a PCR product of the expected size, and restriction enzyme digestion verified successful construction of the recombinant plasmid pGEX-6p-1-E120R (Figure 1C and Figure S1A,B). This result provided the basis for subsequent recombinant expression of GST-tagged pE120R. The plasmid map illustrating the GST fusion framework and insertion sites is shown in Figure 1D. The recombinant plasmid was transformed into *E. coli* Rosetta (DE3), and protein expression was induced with IPTG. SDS-PAGE analysis demonstrated efficient expression of the recombinant protein at the expected molecular weight (55 kDa). Fractionation analysis indicated that the majority of the recombinant protein was present in the soluble supernatant fraction rather than in the inclusion bodies (Figure 1E and Figure S1C).

The fusion protein was subsequently purified by glutathione affinity chromatography. Coomassie blue staining of the eluted fractions revealed strong enrichment of the target band, and Western blot analysis confirmed enrichment and identity of the purified fusion protein (Figure 1F and Figure S1D). Together, these results demonstrate that pE120R could be obtained as a soluble and specifically verified recombinant antigen, supporting its use for monoclonal antibody production.

### 3.2. Immunogenicity Determination of the Recombinant GST-pE120R Protein

**Figure 2:**
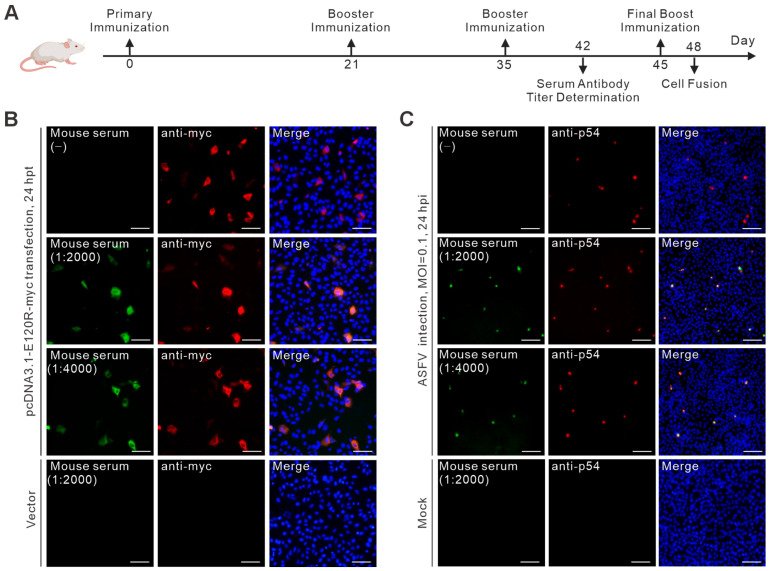
Immunization protocol in mice and determination of serum antibody titers. (**A**) Schematic representation of the mouse immunization procedure using purified GST-pE120R protein, including primary immunization, booster injections, serum collection, and final boosting before cell fusion. (**B**) Serum antibody titers determined by indirect immunofluorescence assay (IFA) in WSL-R4 cells 24 h after transfection with pcDNA3.1-E120R-myc. Scale bar = 100 μm. (**C**) Serum antibody titers determined by IFA in WSL-R4 cells 24 h after ASFV infection at an MOI of 0.1. Scale bar = 200 μm. Green indicates the fluorescence signal of mouse serum, red indicates the signal of the reference antibody (anti-myc in panel (**B**) and anti-p54 in panel (**C**)), and blue indicates nuclei stained with DAPI. In the merged images, overlapping green and red signals appear yellow/orange, indicating co-localization in the same cells.

To determine the immunogenicity of the constructed recombinant protein, mice were immunized with purified GST-pE120R according to the schedule illustrated in Figure 2A. Following the primary immunization, two booster injections were administered, and serum samples were then collected for antibody titer determination. Indirect immunofluorescence assay (IFA) was performed under both plasmid transfection and ASFV infection conditions to assess the reactivity of the immune sera to pE120R in different experimental settings.

In WSL-R4 cells transfected with pcDNA3.1-E120R-myc, no fluorescence signal was detected with negative serum, whereas specific fluorescence signals were observed with immune sera diluted at 1:2000 and 1:4000 (Figure 2B). The staining pattern overlapped with that detected by the anti-Myc antibody, indicating that the immune sera specifically recognized ectopically expressed pE120R. Similarly, in ASFV-infected WSL-R4 cells, immune sera at both dilutions produced distinct fluorescence signals, with a distribution pattern consistent with that of the viral structural protein p54 (Figure 2C). No signal was observed in the negative serum control or the corresponding negative control groups under either transfection or infection conditions. These results indicate that immunization with recombinant GST-pE120R elicited a specific humoral response capable of recognizing pE120R under both transfection and infection conditions. The detectable reactivity at a serum dilution of 1:4000 further supports the suitability of this immunization strategy for subsequent hybridoma fusion and monoclonal antibody generation.

### 3.3. Functional Characterization and Isotype Identification of pE120R Monoclonal Antibodies

**Figure 3:**
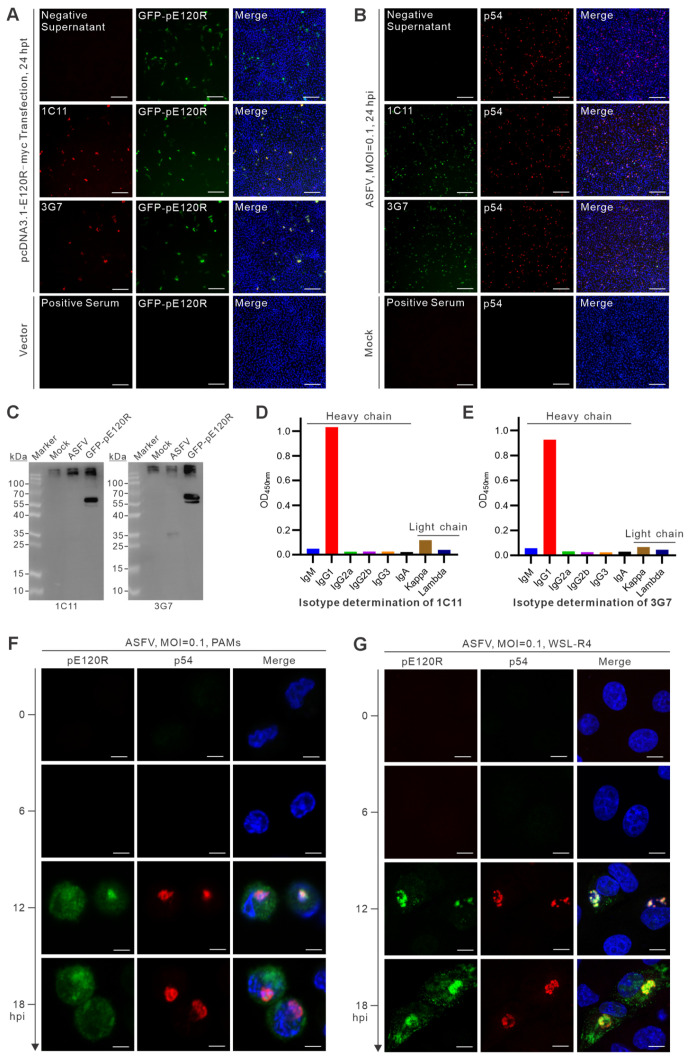
Functional characterization, isotype determination, and localization analysis of pE120R monoclonal antibodies. (**A**) IFA detection of monoclonal antibody titers in WSL-R4 cells 24 h after transfection with pEGFP-C1-E120R. “Negative supernatant” refers to the culture supernatant of hybridoma cells that did not produce antibodies against pE120R, whereas “positive serum” refers to the serum collected from the immunized mouse before cell fusion. Red indicates the fluorescence signal of the tested monoclonal antibodies or positive serum, green indicates GFP-pE120R, and blue indicates nuclei stained with DAPI. Merged images show the overlay of the fluorescence channels. (**B**) IFA detection of antibody reactivity in ASFV-infected WSL-R4 cells (an MOI of 0.1, 24 hpi). Scale bar = 250 μm. Green indicates the fluorescence signal of the tested monoclonal antibodies, red indicates p54 staining, and blue indicates nuclei stained with DAPI. Merged images show the overlay of the fluorescence channels. (**C**) Western blot analysis of pE120R recognition by monoclonal antibodies 1C11 and 3G7. (**D**,**E**) Isotype determination of monoclonal antibodies 1C11 and 3G7. (**F**,**G**) Expression dynamics and subcellular localization of ASFV pE120R during infection analyzed by confocal microscopy. pE120R was detected using monoclonal antibody 3G7. Scale bar = 10 μm.

IFA was first performed to evaluate the reactivity of ascites-derived monoclonal antibodies. In WSL-R4 cells transfected with pEGFP-C1-E120R for 24 h, both 1C11 and 3G7 produced strong fluorescence signals that overlapped with GFP-pE120R expression, whereas no signal was observed with the negative supernatant, indicating that both monoclonal antibodies specifically recognize exogenously expressed pE120R (Figure 3A). Similarly, under ASFV infection conditions (an MOI of 0.1, 24 hpi), both antibodies detected specific fluorescence signals in WSL-R4 cells, with spatial distributions consistent with p54 staining, supporting their applicability for detecting endogenous pE120R in infected cells by immunofluorescence (Figure 3B). However, Western blot analysis revealed that 1C11 and 3G7 specifically detected exogenously expressed GFP-pE120R but failed to recognize pE120R produced during viral infection (Figure 3C and Figure S2A,B). This result indicates that the performance of these antibodies is assay-dependent and that their utility differs between immunofluorescence and immunoblotting applications. Isotype analysis showed that both monoclonal antibodies belong to the IgG1 heavy-chain subclass with κ light chains (Figure 3D,E).

Confocal immunofluorescence microscopy was further utilized to analyze the temporal expression pattern and subcellular localization of pE120R during infection. In both infected porcine alveolar macrophages (PAMs) and WSL-R4 cells, no pE120R signal was detected during the early stages of infection, whereas clear expression emerged at the mid-to-late stages and showed partial colocalization with p54, suggesting that pE120R accumulates in viral factory-associated regions during the late phase of infection (Figure 3F,G).

### 3.4. Epitope Mapping of pE120R Monoclonal Antibodies

**Figure 4:**
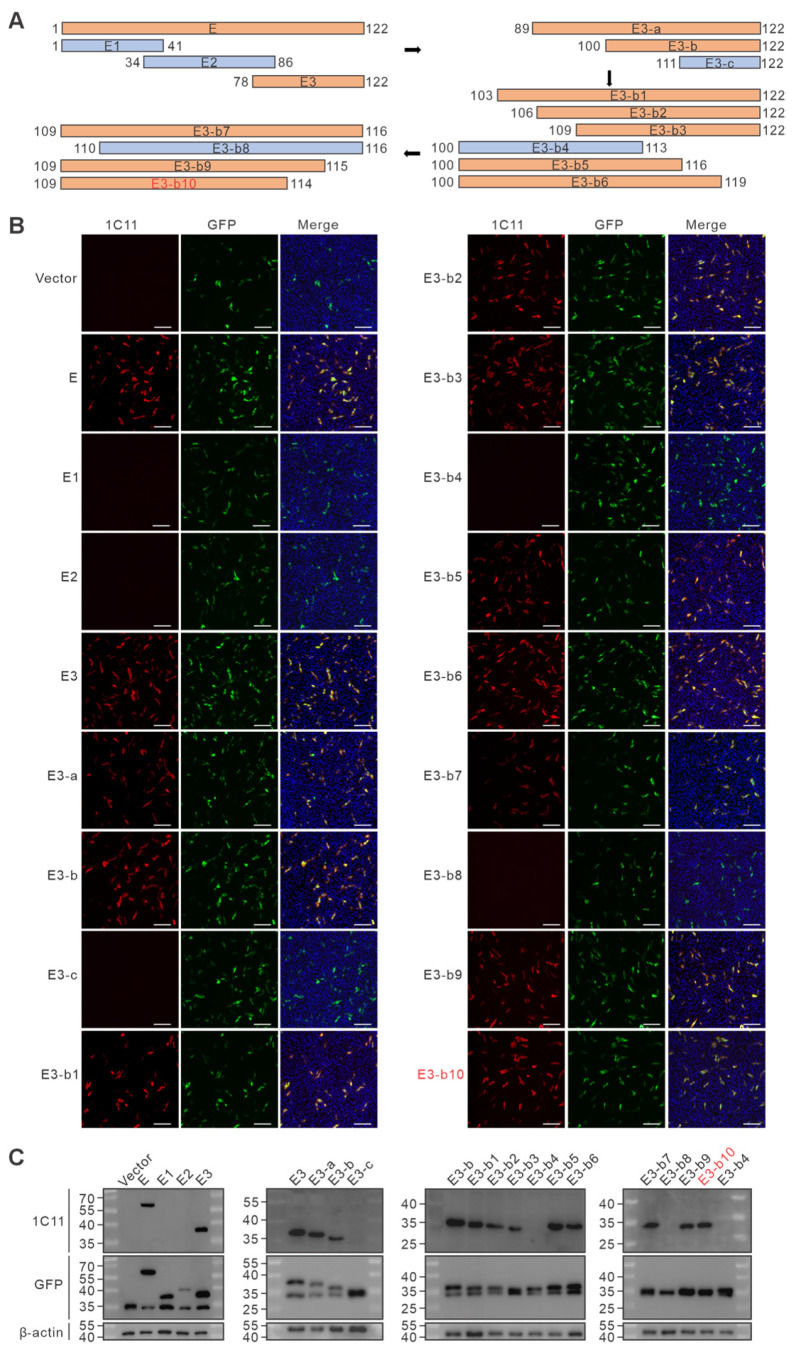
Epitope mapping of monoclonal antibody 1C11 against pE120R. (**A**) Schematic illustration of GFP-tagged truncation constructs of pE120R. Black arrows indicate the direction of stepwise epitope mapping and progressive narrowing of the truncation range. Orange regions indicate fragments recognized by 1C11, whereas blue regions indicate non-reactive fragments. Red text indicates the key truncation construct used to define the minimal linear epitope. Numbers denote amino acid positions. (**B**) Indirect immunofluorescence assay (IFA) detection of 1C11 reactivity in cells transfected with pE120R truncation constructs. Red indicates the fluorescence signal of 1C11, green indicates GFP-tagged truncation constructs, blue indicates nuclei stained with DAPI, and Merge shows the overlay of the fluorescence channels. Scale bar = 250 μm. (**C**) Western blot analysis of 1C11 binding to pE120R truncation constructs expressed in transfected cells.

**Figure 5:**
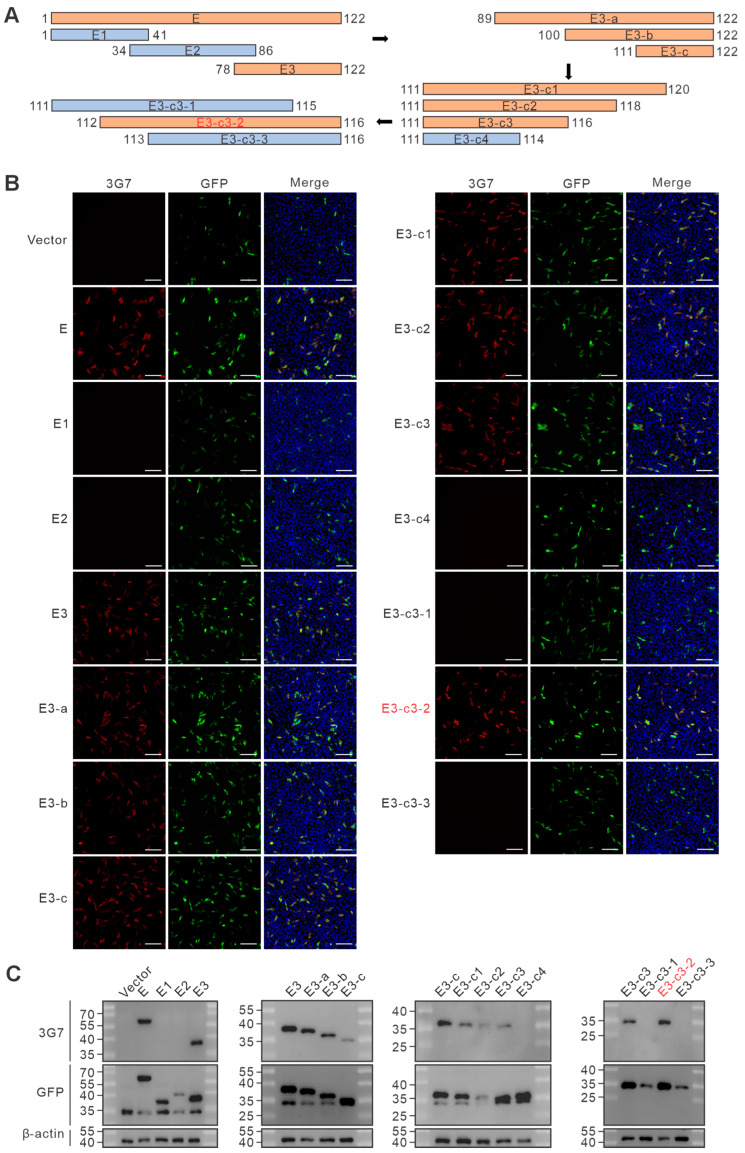
Epitope mapping of monoclonal antibody 3G7 against pE120R. (**A**) Schematic illustration of GFP-tagged truncation constructs of pE120R. Black arrows indicate the direction of stepwise epitope mapping and progressive narrowing of the truncation range. Orange regions indicate fragments recognized by 3G7, whereas blue regions indicate non-reactive fragments. Red text indicates the key truncation construct used to define the minimal linear epitope. Numbers denote amino acid positions. (**B**) Indirect immunofluorescence assay (IFA) detection of 3G7 reactivity in cells transfected with pE120R truncation constructs. Red indicates the fluorescence signal of 3G7, green indicates GFP-tagged truncation constructs, blue indicates nuclei stained with DAPI, and Merge shows the overlay of the fluorescence channels. Scale bar = 250 μm. (**C**) Western blot analysis of 3G7 binding to pE120R truncation constructs expressed in transfected cells.

Epitope identification of monoclonal antibodies 1C11 and 3G7 was performed using a series of truncated, GFP-tagged pE120R expression constructs. After transfection, cells were analyzed by Western blot and indirect immunofluorescence assay (IFA). After four rounds of screening, the epitope recognized by 1C11 was identified as ^109^KKHLFP^114^ (Figure 4 and Figure S3), whereas that recognized by 3G7 was mapped to ^112^LFPKL^116^ (Figure 5 and Figure S4). The results indicate that both monoclonal antibodies recognize linear epitopes located in the C-terminal region of pE120R. The partial overlap between the two mapped epitopes further suggests that this region may represent an antigenically prominent site within the protein, providing a structural basis for subsequent analyses of pE120R antigenicity and antibody recognition.

### 3.5. Structural and Conservation Analysis of Epitopes Recognized by pE120R Monoclonal Antibodies

**Figure 6:**
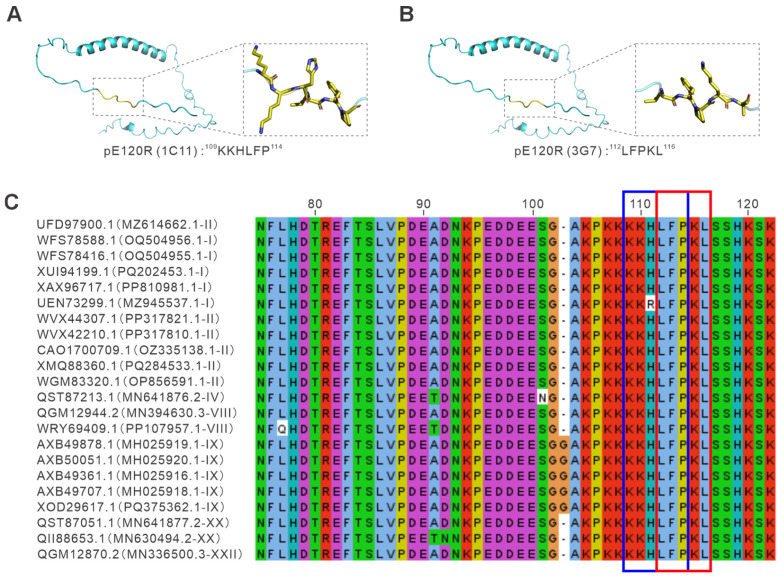
Structural localization and conservation analyses of epitopes recognized by monoclonal antibodies 1C11 and 3G7. (**A**,**B**) Structural mapping of the epitopes on the predicted three-dimensional model of ASFV pE120R generated by AlphaFold. Epitope residues were visualized and highlighted using PyMOL (version 3.10). Cyan indicates the overall predicted structure of pE120R, yellow indicates the epitope residues. (**A**) The epitope recognized by mAb 1C11 (^109^KKHLFP^114^) is shown in yellow, together with a magnified view of the side-chain orientation. (**B**) The epitope recognized by mAb 3G7 (^112^LFPKL^116^) is displayed in the same manner. Both epitopes are located in the C-terminal region and exposed on the protein surface. (**C**) Multiple sequence alignment of the C-terminal region of pE120R from representative ASFV strains using CADC_HN09 (GenBank: MZ614662.1) as the reference sequence. Labels on the left indicate the GenBank protein accession number followed by the corresponding ASFV strain accession number and genotype. Different letter colors indicate different amino acid residue types in the alignment. The blue box indicates residues 109–114, and the red box indicates residues 112–116, corresponding to the mapped epitope regions. Conserved residues are indicated by identical coloring across the aligned sequences.

To further determine the spatial distribution of the epitopes recognized by monoclonal antibodies 1C11 and 3G7 within the protein, the three-dimensional structure of ASFV pE120R was predicted using AlphaFold and visualized with PyMOL (version 3.10). The model indicated that pE120R is predominantly composed of α-helical elements, whereas its C-terminal region adopts a flexible disordered coil conformation. Epitope mapping showed that the epitopes recognized by 1C11 and 3G7 are both located on the surface of the C-terminus (Figure 6A,B), indicating that this region is structurally accessible to antibody binding and supporting the experimentally mapped linear epitope profile.

Furthermore, to assess the conservation of the linear epitopes recognized by mAbs 1C11 and 3G7, sequence analyses were performed across multiple ASFV strains. Using ASFV CADC_HN09 as the reference strain, multiple sequence alignment of pE120R amino acid sequences revealed that the C-terminal region encompassing residues 109–116 is highly conserved among different isolates (Figure 6C). Only minor amino acid substitutions were observed in a limited number of strains, while the core conserved motif remained largely unchanged. Taken together, these results indicate that the epitopes identified for 1C11 and 3G7 are not only surface-accessible but also evolutionarily conserved, which may underlie their potential value for the comparative detection of different ASFV strains and for future studies of pE120R function.

## 4. Discussion

African swine fever virus (ASFV) encodes numerous structural and regulatory proteins that remain incompletely characterized [21,22,23], limiting mechanistic studies of virion morphogenesis and host interactions. pE120R is a late gene product previously implicated in particle assembly and trafficking through interaction with the major capsid protein p72 and the cell cytoskeleton [15,24]. Therefore, the development of specific tools targeting pE120R is important for investigating its expression pattern and subcellular localization during ASFV infection. Here, we developed monoclonal antibodies against pE120R to enable detection and localization studies in relevant cellular contexts.

Using bacterially expressed and purified GST-tagged pE120R as the immunogen, we generated two pE120R-specific mAbs, 1C11 and 3G7, both belonging to the IgG1-κ isotype. Indirect immunofluorescence assays demonstrated that both antibodies specifically recognized pE120R in transfected cells as well as in ASFV-infected cells, indicating that these mAbs are suitable for monitoring pE120R expression and subcellular distribution during infection. In infected cells, pE120R became detectable during the mid-to-late stages of infection and exhibited partial colocalization with p54, a commonly used marker of ASFV viral factories (VFs) [25]. Given that VFs are the principal sites of ASFV genome replication and virion assembly [26], this spatial distribution is consistent with the localization of pE120R to VF-associated regions where morphogenesis and early intracellular trafficking occur.

A notable finding of this study was the assay-dependent performance of the two mAbs. Although both antibodies robustly detected ectopically expressed pE120R by Western blot, no specific band corresponding to pE120R was detected in ASFV-infected cell lysates under denaturing conditions. This difference may be attributable to both biological and technical factors. Specifically, the abundance of endogenous pE120R during infection is likely much lower than that achieved in overexpression systems, thereby reducing the sensitivity of Western blot detection [27]. In addition, overexpressed proteins often exist in a relatively free form, whereas endogenous proteins under infection conditions may be present in protein complexes or assembly-associated states, which could make their epitopes less accessible during Western blot processing [28,29]. Meanwhile, immunoblotting depends on sufficient recovery of total antigen from whole-cell lysates, and the final signal may also be influenced by lysis efficiency, protein extraction efficiency, and membrane transfer efficiency. In contrast, IFA allows visualization of locally enriched antigen within infected cells, particularly in viral factory-associated regions. Taken together, these findings highlight the importance of validating ASFV antibodies for their intended applications and also caution against inferring antigen abundance solely from immunofluorescence intensity.

Epitope mapping using GFP-tagged truncation constructs localized the binding sites of 1C11 and 3G7 to residues ^109^KKHLFP^114^ and ^112^LFPKL^116^, respectively, revealing an overlapping linear epitope cluster in the C-terminal region of pE120R. The fact that two independently derived mAbs recognized closely adjacent sequences suggests that this region may represent an antigenically prominent site of pE120R. Structural interpretation using AlphaFold-predicted models suggested that this region is flexible and surface-exposed, consistent with the behavior of linear B-cell epitopes [30] and with the strong performance of both mAbs in immunofluorescence assays. Furthermore, sequence alignment across representative ASFV isolates indicated that this C-terminal segment is highly conserved, supporting that it may be subject to evolutionary constraint and could be important for maintaining pE120R structure or function. Given that pE120R has been implicated in virion assembly, intracellular transport, and viral factory-associated localization, the identification of a conserved and exposed epitope cluster at its C-terminus raises the possibility that this region may participate in protein–protein interactions relevant to viral morphogenesis or trafficking. Although this study does not directly address the functional role of this C-terminal segment, the mapped epitopes provide a useful framework for future studies aimed at dissecting how pE120R contributes to ASFV replication and viral biology.

Overall, the mAbs generated in this study provide practical tools for studying pE120R expression and localization, and define a conserved, surface-accessible epitope region that will facilitate future studies on the antigenic properties and molecular functions of this protein. Their ability to recognize pE120R in virus-related assays also supports their potential utility in mechanistic studies, including tracking the viral factory-associated localization of pE120R, probing its involvement in virion assembly and intracellular trafficking, and characterizing its interactions with viral or host factors. In addition, the identified epitope information may be valuable for the development of diagnostic reagents and for antigen design in future ASFV-related immunological studies. Nevertheless, this study has several limitations, including the lack of high-resolution structural validation of antibody–antigen interactions; the absence of functional assays such as neutralization, viral replication interference, or diagnostic validation; and the limited ability of these antibodies to detect endogenous pE120R in Western blot assays. These monoclonal antibodies will be applied in future studies to investigate the roles of pE120R in ASFV replication, intracellular trafficking, and immune regulation. Combined with interactome approaches and structural analyses, such as cryo-EM, these efforts may further clarify how pE120R participates in virion assembly and intracellular trafficking-related pathways during ASFV infection.

## 5. Conclusions

In this study, two monoclonal antibodies (1C11 and 3G7) specifically targeting ASFV pE120R were successfully generated and characterized, both belonging to the IgG1-κ isotype. Functional evaluation and epitope mapping demonstrated their applicability in transfection- and infection-related detection assays and precisely localized their binding epitopes to residues ^109^KKHLFP^114^ and ^112^LFPKL^116^ within the C-terminal region of pE120R. Structural prediction further indicated that these epitopes are surface-exposed, providing spatial insights into antibody–antigen recognition. Importantly, these antibodies not only expand the available reagent toolbox for ASFV research, but also provide useful tools for monitoring pE120R expression, studying its viral factory-associated localization, and supporting future investigations into its molecular function during infection. In addition, the defined epitope information may facilitate the development of diagnostic reagents and contribute to antigen design for future ASFV-related studies. Collectively, the monoclonal antibodies and epitope information established here provide valuable experimental tools for future investigations into the molecular functions of pE120R during ASFV infection and lay a foundation for subsequent functional and diagnostic studies.

## Figures and Tables

**Table 1:** Primers for pE120R truncation constructs.

Plasmid Name	Primer Sequence * (5′–3′)	
pGEX-6p-1-E120R	F	ttccaggggcccctgggatccATGGCAGATTTTAATTCTCCAATCC
	R	gtcacgatgcggccgctcgagTTACTTCGATTTATGCGAGCTTAATT
pEGFP-E120R(1–122aa)	F	aattctgcagtcgacggtaccATGGCAGATTTTAATTCTCCAATCC
	R	ttatctagatccggtggatccTTACTTCGATTTATGCGAGCTTAATT
pEGFP-E120R(1–41aa)	F	aattctgcagtcgacggtaccATGGCAGATTTTAATTCTCCAATCC
	R	ttatctagatccggtggatccTTACAAGCCTGCTGCGAAGCTC
pEGFP-E120R(34–86aa)	F	aattctgcagtcgacggtaccATGATACCGAGCTTCGCAGCAGG
	R	ttatctagatccggtggatccTTATGAAGTAAACTCCCTAGTATCGT
pEGFP-E120R(78–122aa)	F	aattctgcagtcgacggtaccATGCTACACGATACTAGGGAGTTTAC
	R	ttatctagatccggtggatccTTACTTCGATTTATGCGAGCTTAATT
pEGFP-E120R(89–122aa)	F	aattctgcagtcgacggtaccATGCCCGATGAGGCAGACAATAAA
	R	ttatctagatccggtggatccTTACTTCGATTTATGCGAGCTTAATT
pEGFP-E120R(100–122aa)	F	aattctgcagtcgacggtaccATGGAAGAAAGCGGTGCAAAACCT
	R	ttatctagatccggtggatccTTACTTCGATTTATGCGAGCTTAATT
pEGFP-E120R(111–122aa)	F	aattctgcagtcgacggtaccATGCATTTGTTTCCAAAATTAAGC
	R	ttatctagatccggtggatccTTACTTCGATTTATGCGAGCTTAATT
pEGFP-E120R(103–122aa)	F	aattctgcagtcgacggtaccATGGGTGCAAAACCTAAAAAG
	R	ttatctagatccggtggatccTTACTTCGATTTATGCGAGCTTAATT
pEGFP-E120R(106–122aa)	F	aattctgcagtcgacggtaccATGCCTAAAAAGAAAAAACAT
	R	ttatctagatccggtggatccTTACTTCGATTTATGCGAGCTTAATT
pEGFP-E120R(109–122aa)	F	aattctgcagtcgacggtaccATGAAAAAACATTTGTTTCCA
	R	ttatctagatccggtggatccTTACTTCGATTTATGCGAGCTTAATT
pEGFP-E120R(100–113aa)	F	aattctgcagtcgacggtaccATGGAAGAAAGCGGTGCAAAA
	R	ttatctagatccggtggatccTTAAAACAAATGTTTTTTCTT
pEGFP-E120R(100–116aa)	F	aattctgcagtcgacggtaccATGGAAGAAAGCGGTGCAAAA
	R	ttatctagatccggtggatccTTATAATTTTGGAAACAAATG
pEGFP-E120R(100–119aa)	F	aattctgcagtcgacggtaccATGGAAGAAAGCGGTGCAAAA
	R	ttatctagatccggtggatccTTAATGCGAGCTTAATTTTGG
pEGFP-E120R(109–116aa)	F	aattctgcagtcgacggtaccATGAAAAAACATTTGTTTCCA
	R	ttatctagatccggtggatccTTATAATTTTGGAAACAAATG
pEGFP-E120R(110–116aa)	F	ttatctagatccggtggatccTTATTTTTTCTTTTTAGGTTT
	R	ttatctagatccggtggatccTTATAATTTTGGAAACAAATG
pEGFP-E120R(109–115aa)	F	aattctgcagtcgacggtaccATGAAAAAACATTTGTTTCCA
	R	ttatctagatccggtggatccTTATTTTGGAAACAAATGTTTTTT
pEGFP-E120R(109–114aa)	F	aattctgcagtcgacggtaccATGAAAAAACATTTGTTTCCA
	R	ttatctagatccggtggatccTTATGGAAACAAATGTTTTTT
pEGFP-E120R(111–120aa)	F	aattctgcagtcgacggtaccATGCATTTGTTTCCAAAATTAAGCT
	R	ttatctagatccggtggatccTTATTTATGCGAGCTTAATTTTGGA
pEGFP-E120R(111–118aa)	F	aattctgcagtcgacggtaccATGCATTTGTTTCCAAAATTAAGCT
	R	ttatctagatccggtggatccTTACGAGCTTAATTTTGGAAACAAA
pEGFP-E120R(111–116aa)	F	aattctgcagtcgacggtaccATGCATTTGTTTCCAAAATTA
	R	ttatctagatccggtggatccTTATAATTTTGGAAACAAATG
pEGFP-E120R(111–114aa)	F	aattctgcagtcgacggtaccATGCATTTGTTTCCA
	R	ttatctagatccggtggatccTTATGGAAACAAATG
pEGFP-E120R(111–115aa)	F	aattctgcagtcgacggtaccATGCATTTGTTTCCAAAA
	R	ttatctagatccggtggatccTTATTTTGGAAACAAATG
pEGFP-E120R(112–116aa)	F	aattctgcagtcgacggtaccATGTTGTTTCCAAAATTA
	R	ttatctagatccggtggatccTTATAATTTTGGAAACAA
pEGFP-E120R(113–116aa)	F	aattctgcagtcgacggtaccATGTTTCCAAAATTA
	R	ttatctagatccggtggatccTTATAATTTTGGAAA

* In the primer sequences, uppercase letters denote sequences complementary to the target gene, whereas lowercase letters denote overlapping sequences designed for homologous recombination with the vector.

## Data Availability

The original contributions presented in this study are included in the article/Supplementary Material. Further inquiries can be directed to the corresponding author.

## Supplementary Materials

The following supporting information can be downloaded at: https://www.mdpi.com/article/10.3390/vetsci13040358/s1. Figure S1: Original data for the expression and purification of pE120R protein; Figure S2: Original Western blot images showing recognition of pE120R by monoclonal antibodies 1C11 and 3G7; Figure S3: Original Western blot data for epitope mapping with monoclonal antibody 1C11; Figure S4: Original Western blot data for epitope mapping with monoclonal antibody 3G7.
