# Supplementary material for: Preparation and Epitope Identification of Monoclonal Antibodies Against African Swine Fever Virus pE120R
Source: Vet Sci. 2026 Apr 7;13(4):358. doi: 10.3390/vetsci13040358 (PMC13120246; doi:10.3390/vetsci13040358)
Supplement: Supplementary file 1 [file vetsci-13-00358-s001.zip › vetsci-4207942-supplementary.pdf]

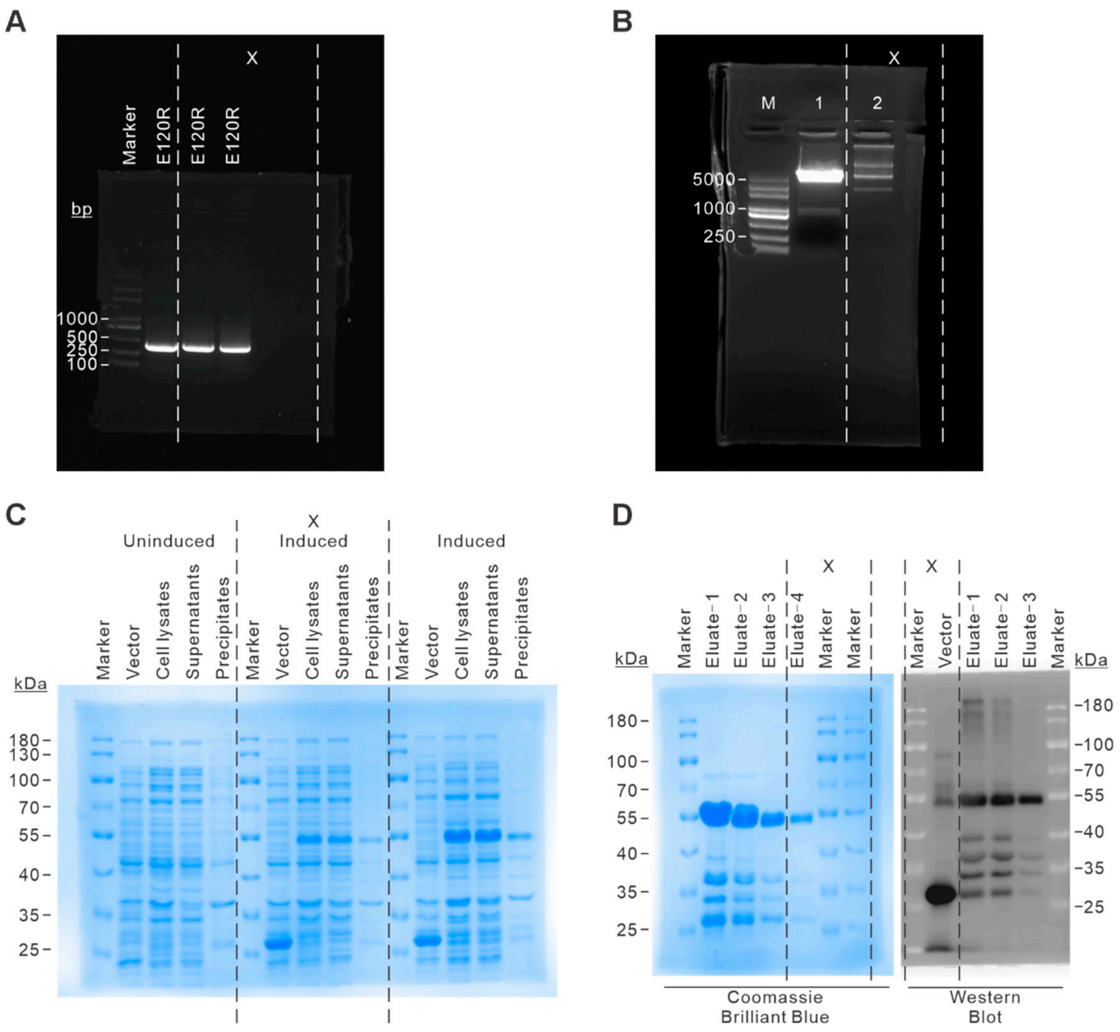

Figure S1. Original data for the expression and purification of pE120R protein. (A) PCR amplification of the E120R gene. (B) Double-enzyme digestion of the pGEX-6p-1 vector; lane 1, pGEX-6p-1 linearized by BamHI/XhoI digestion; lane 2, undigested pGEX-6p-1 vector. (C) SDS–PAGE analysis of GST-pE120R expression in *Escherichia coli* under uninduced and induced conditions. (D) SDS–PAGE and Western blot analysis of purified GST-pE120R protein. Eluted fractions were resolved by SDS–PAGE and visualized by Coomassie Brilliant Blue staining (left) or detected by Western blotting using an anti-GST antibody (right). Regions marked with X denote portions of the original gel or blot images that were not included in the corresponding figure shown in the main text.

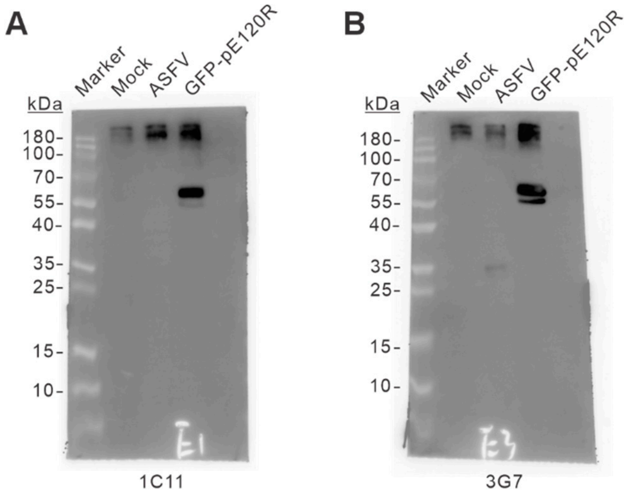

Figure S2. Original Western blot images showing recognition of pE120R by monoclonal antibodies 1C11(A) and 3G7(B).

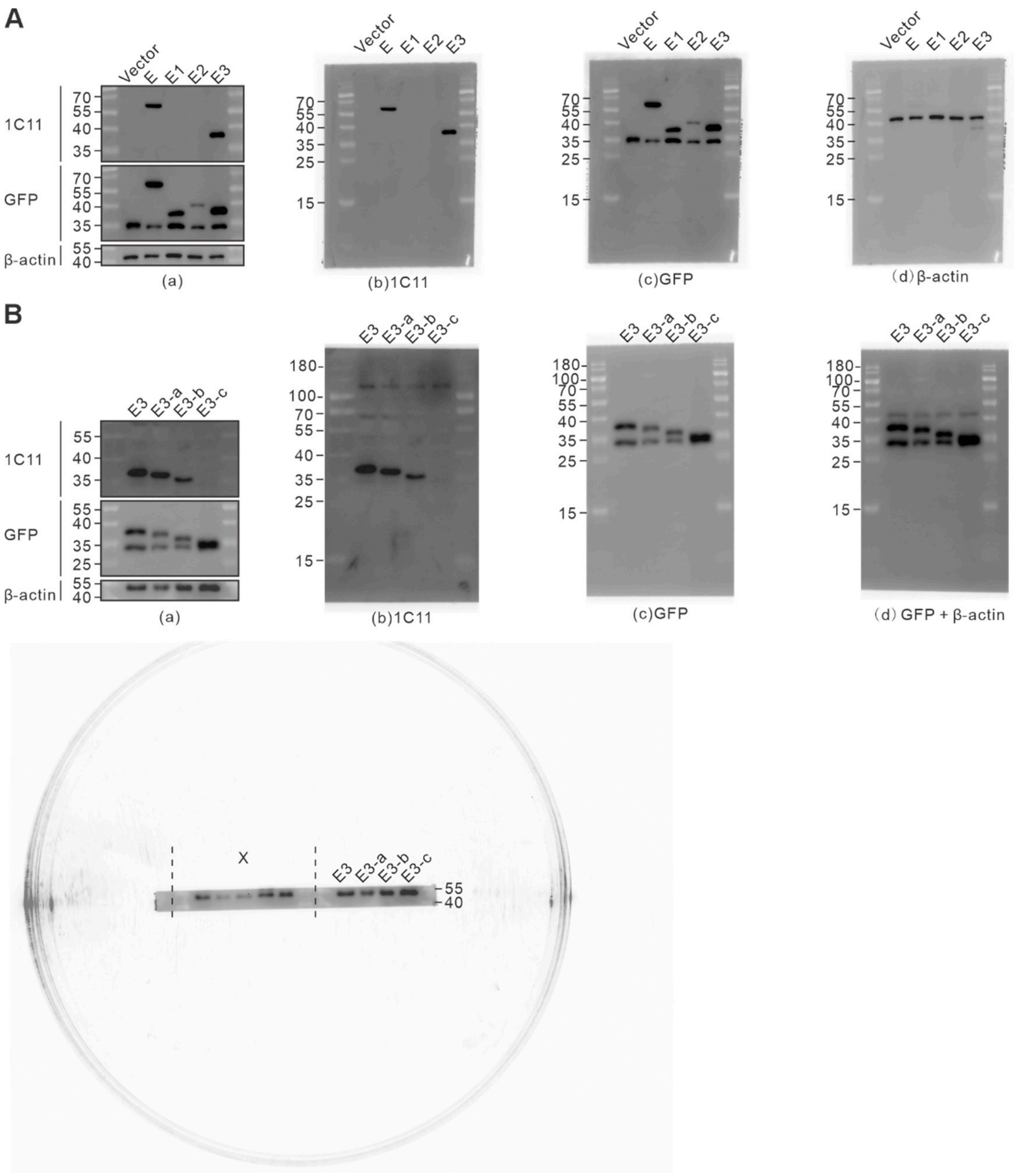
A
Vector E E1 E2 E3
1C11
GFP
β-actin
70 55 40 35
70 55 40 35
55 40
(a)
70 55 40 35 25 15
(b)1C11
(c)GFP
(d)β-actin
B
E3 E3-a E3-b E3-c
1C11
GFP
β-actin
55 40 35
55 40 35 25
55 40
(a)
180 100 70 55 40 35 25 15
(b)1C11
(c)GFP
(d) GFP + β-actin
X
E3 E3-a E3-b E3-c
55
40

(e)β-actin

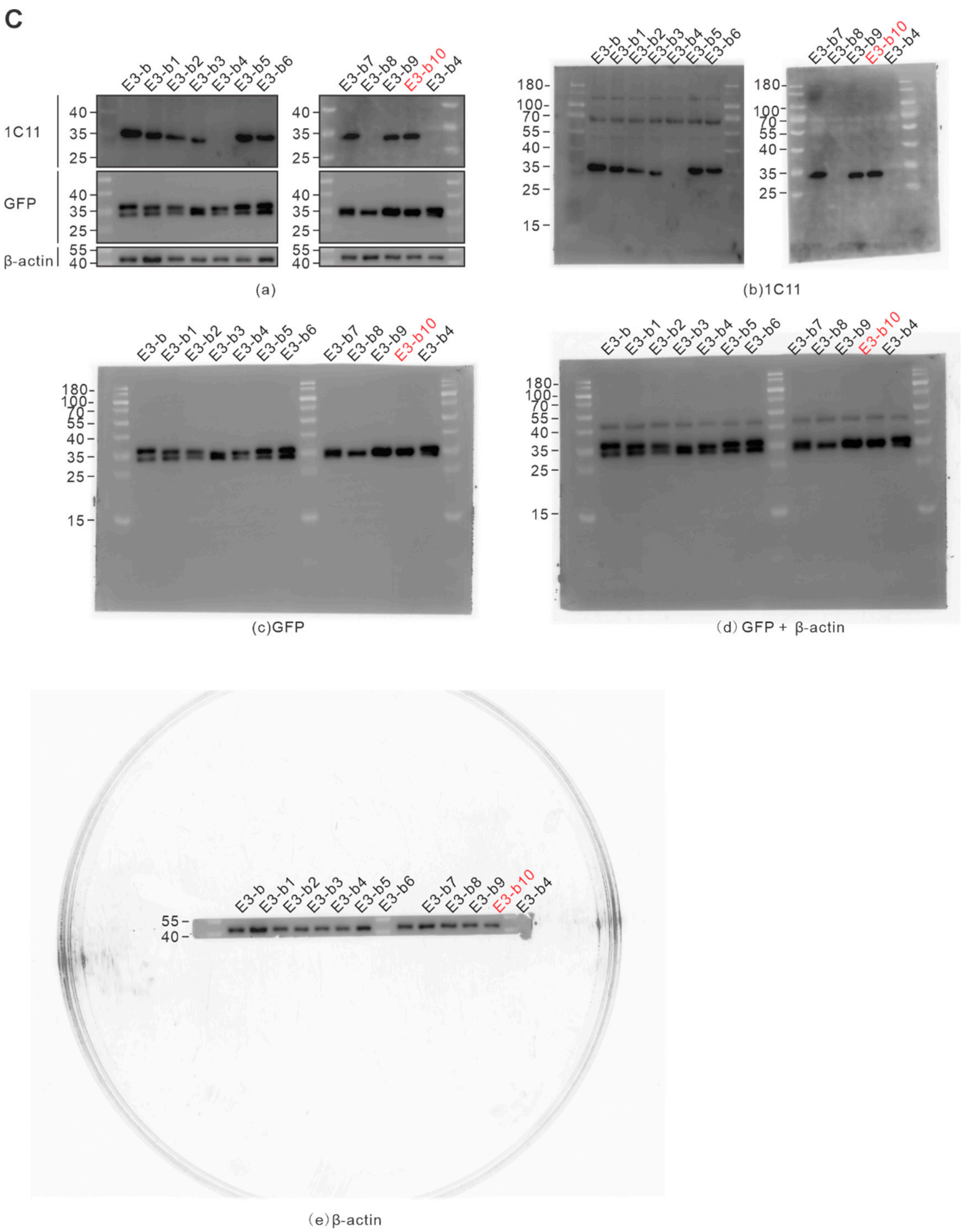

Figure S3. Original western blot data for epitope mapping with monoclonal antibody 1C11.

(A–C) Western blot results from rounds 1, 2, and 3–4 of epitope mapping, respectively. In each panel, (a) shows the cropped/processed blot image presented in the main text, whereas (b–e) show the corresponding original western blot data. Blots were incubated with 1C11, anti-GFP, or anti-β-actin, as indicated in the figure. 1C11 was used for epitope identification, anti-GFP was used to confirm the expression of the recombinant truncation fragments, and β-actin served as a loading control.

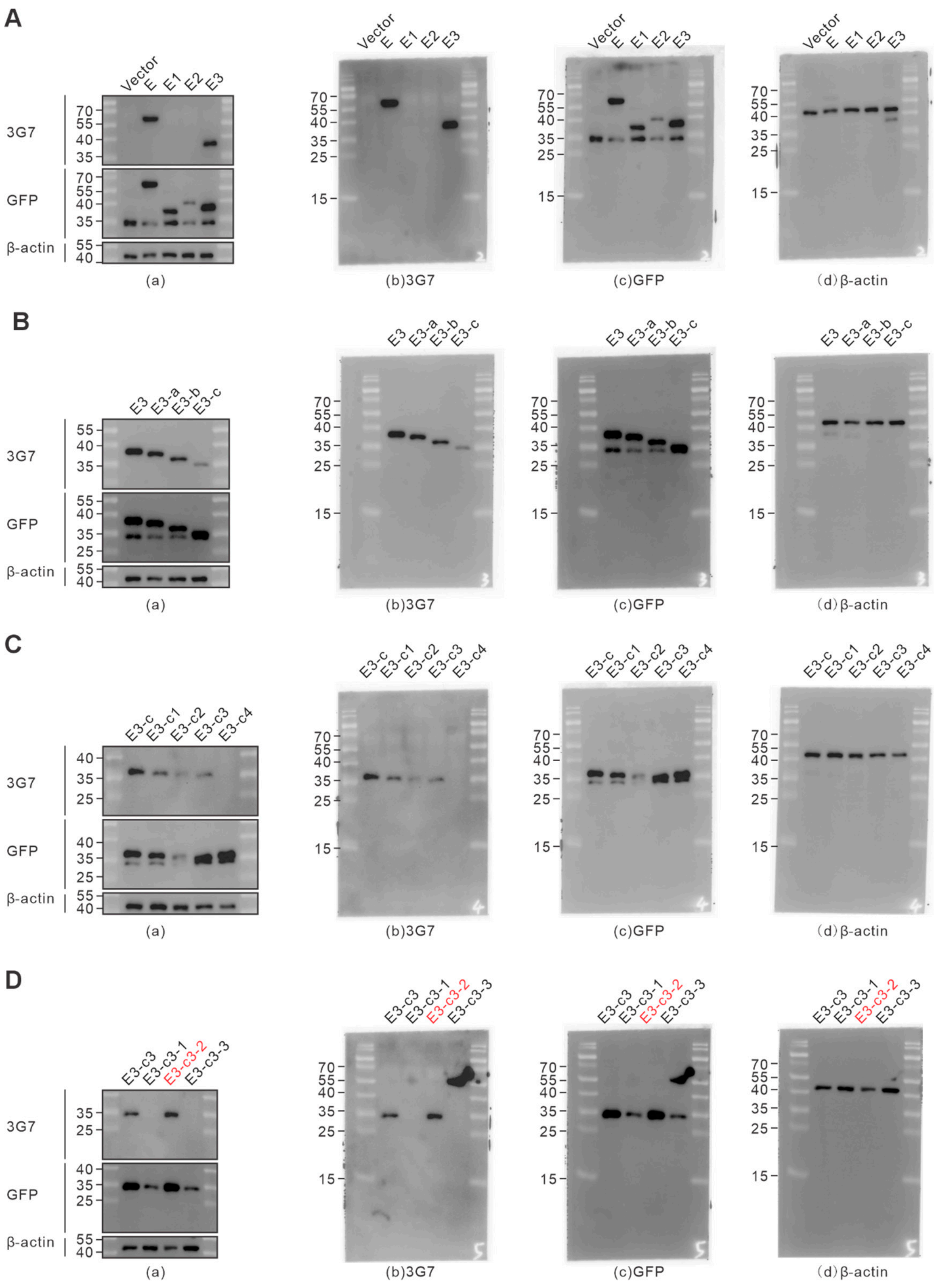

Figure S4. Original western blot data for epitope mapping with monoclonal antibody 3G7. (A–D) Western blot results from rounds 1, 2, 3, and 4 of epitope mapping, respectively. In each panel, (a) shows the cropped/processed blot image presented in the main text, whereas (b–d) show the corresponding original western blot data. Blots were incubated with 3G7, anti-GFP, or anti-β-actin, as indicated in the figure. 3G7 was used for epitope identification, anti-GFP was used to confirm the expression of the recombinant truncation fragments, and β-actin served as a loading control.
